# SARS-CoV-2 ORF8 Forms Intracellular Aggregates and Inhibits IFNγ-Induced Antiviral Gene Expression in Human Lung Epithelial Cells

**DOI:** 10.3389/fimmu.2021.679482

**Published:** 2021-06-09

**Authors:** Hua Geng, Saravanan Subramanian, Longtao Wu, Heng-Fu Bu, Xiao Wang, Chao Du, Isabelle G. De Plaen, Xiao-Di Tan

**Affiliations:** ^1^ Center for Intestinal and Liver Inflammation Research, Division of Pediatric Gastroenterology, Hepatology and Nutrition, Department of Pediatrics, Ann and Robert H. Lurie Children’s Hospital of Chicago, Chicago, IL, United States; ^2^ Department of Pediatrics, Feinberg School of Medicine, Northwestern University, Chicago, IL, United States; ^3^ Section of Neurosurgery, Department of Surgery, University of Chicago, Chicago, IL, United States; ^4^ Division of Neonatology, Department of Pediatrics, Ann & Robert H. Lurie Children’s Hospital of Chicago, Chicago, IL, United States; ^5^ Department of Pathology, Feinberg School of Medicine, Northwestern University, Chicago, IL, United States; ^6^ Research Service, Jesse Brown Veterans Affairs Medical Center, Chicago, IL, United States

**Keywords:** SARS-CoV-2 accessory protein, ORF8, lung epithelial cells, interferon signaling, inflammation

## Abstract

Infection with the severe acute respiratory syndrome coronavirus 2 (SARS-CoV-2) causes COVID-19, a disease that involves significant lung tissue damage. How SARS-CoV-2 infection leads to lung injury remains elusive. The open reading frame 8 (ORF8) protein of SARS-CoV-2 (ORF8^SARS-CoV-2^) is a unique accessory protein, yet little is known about its cellular function. We examined the cellular distribution of ORF8^SARS-CoV-2^ and its role in the regulation of human lung epithelial cell proliferation and antiviral immunity. Using live imaging and immunofluorescent staining analyses, we found that ectopically expressed ORF8^SARS-CoV-2^ forms aggregates in the cytosol and nuclear compartments of lung epithelial cells. Using *in silico* bioinformatic analysis, we found that ORF8^SARS-CoV-2^ possesses an intrinsic aggregation characteristic at its N-terminal residues 1-18. Cell culture did not reveal any effects of ORF8^SARS-CoV-2^ expression on lung epithelial cell proliferation and cell cycle progression, suggesting that ORF8^SARS-CoV-2^ aggregates do not affect these cellular processes. Interestingly, ectopic expression of ORF8^SARS-CoV-2^ in lung epithelial cells suppressed basal expression of several antiviral molecules, including DHX58, ZBP1, MX1, and MX2. In addition, expression of ORF8^SARS-CoV-2^ attenuated the induction of antiviral molecules by IFNγ but not by IFNβ in lung epithelial cells. Taken together, ORF8^SARS-CoV-2^ is a unique viral accessory protein that forms aggregates when expressing in lung epithelial cells. It potently inhibits the expression of lung cellular anti-viral proteins at baseline and in response to IFNγ in lung epithelial cells, which may facilitate SARS-CoV-2 escape from the host antiviral innate immune response during early viral infection. In addition, it seems that formation of ORF8^SARS-CoV-2^ aggregate is independent from the viral infection. Thus, it would be interesting to examine whether any COVID-19 patients exhibit persistent *ORF8 SARS-CoV-2* expression after recovering from SARS-CoV-2 infection. If so, the pathogenic effect of prolonged ORF8^SARS-CoV-2^ expression and its association with post-COVID symptoms warrant investigation in the future.

## Introduction

The global coronavirus disease 2019 (COVID-19) pandemic is caused by a novel beta coronavirus, severe acute respiratory syndrome coronavirus 2 (SARS-CoV-2). This new pathogenic virus infects epithelial cells and macrophages in the lungs, which leads to severe inflammation in the respiratory system and diffuse alveolar damage (1–3). However, the key molecular mechanisms leading to lung injury after SARS-CoV-2 infection are not clear. Other pathogenic beta CoVs, including SARS-CoV and MERS-CoV, caused large-scale outbreaks in recent decades (4, 5). Previous studies demonstrated that the genome of CoVs encodes not only a set of viral structural proteins and a replicase complex but also a group of accessory proteins (6) that disrupt host signaling pathways and cell function (7). For example, previous studies found that the SARS-CoV ORF6 protein is present in the endoplasmic reticulum (ER)/Golgi membrane of infected cells (8), where it sequesters nuclear import factors on the rough ER/Golgi membrane and antagonizes STAT1-mediated antiviral signaling (9). Several SARS-CoV accessory proteins, including ORF3a, ORF8a, and ORF8b, have been shown to induce epithelial cell injury (10–13). SARS-CoV ORF8b can activate NLRP3-mediated inflammasome signaling (12). Similarly, MERS-CoV ORF4b binds to regulatory molecules including TBK1 and IKKϵ in cells and modulates interferon (IFN)-β production through inhibition of IRF3 phosphorylation (14). Furthermore, MERS-CoV ORF4b has 2’-5’-phosphodiesterase activity and antagonizes the host anti-viral OAS-RNase L pathway (15). Like other CoVs, the SARS-CoV-2 genome encodes various accessory proteins; however, the pathogenic roles of these proteins are largely unknown.

It has been reported that SARS-CoV and SARS-CoV-2 share a large amount of sequence identity (1). Interestingly, SARS-CoV-2 accessory protein ORF8 (ORF8^SARS-CoV-2^) has a low sequence similarity to SARS-CoV ORF8a, 8b, or 8ab. ORF8^SARS-CoV-2^ protein is highly expressed in patients with SARS-CoV-2 infection (16, 17). Recently, Young et al. (18) observed an association between ORF8^SARS-CoV-2^ and the severity of COVID-19 disease, suggesting that ORF8^SARS-CoV-2^ may play an important role in the pathogenesis of COVID-19. However, little is known about whether ORF8^SARS-CoV-2^ affects the host cell immunological response. Therefore, we investigated the fundamental question of whether and how lung epithelial cells respond to ORF8^SARS-CoV-2^ expression.

## Materials and Methods

### Cell Culture

Human lung epithelial cell line A549 (derived from human pulmonary adenocarcinoma) and human embryonic kidney epithelial cell line HEK293 were obtained from American Type Culture Collection (ATCC, Manassas, VA). A549 and HEK cells were respectively cultured in ATCC-formulated F-12K and DMEM medium supplemented with 10% (v/v) fetal bovine serum, 50 U/ml penicillin, and 50 μg/ml streptomycin at 37°C in a humidified incubator with 5% CO_2_.

### Plasmid Construction and Transfection

The gBlocks gene fragment of ORF8^SARS-CoV-2^ (RefSeq MN908947 27894 to 28256) was synthesized by Integrated DNA Technologies (Coralville, IA) and cloned into a pEGFP-N1 expression vector with C-terminal eGFP fusion, resulting in a new plasmid construct, pEGFP-N1-ORF8. The identity of pEGFP-N1-ORF8 was confirmed by Sanger sequencing. To generate a plasmid construct of ORF8^SARS-CoV-2^ with C-terminal Flag tag, pEGFP-N1-ORF8 was used as a template for PCR amplification and subcloned into pcDNA3.1 BamHI and EcoRI sites using In-Fusion HD Cloning kit (Clontech). The identity of pcDNA3.1-ORF8-Flag construct was confirmed by Sanger sequencing. A549 or HEK293 cells were seeded onto 6-well plates at a density of 3.5 × 10^5^ cells/well and cultured at 37°C for 24 hours before transfection. Cells were then transfected with empty vector pEGFP-N1 or pEGFP-N1-ORF8 plasmids (2.5 µg/well), respectively, using Lipofectamine 3000 (Thermo Fisher Scientific, Waltham, MA) according to the manufacturer’s protocol. In some experiments, cells were co-transfected with a mixture of plasmids (2.5 µg/well) and Poly(I:C) (25 ng/well, InvivoGen, San Diego, CA). Culture medium was refreshed after 4 hours, and cells were processed for analysis 24-48 hours post-transfection. Some cells were treated with IFNβ- or IFNγ-containing medium (100 ng/mL, PeproTech, Rocky Hill, NJ) after transfection and then processed for analysis.

### Protein Extraction and Immunoblotting

Total protein was isolated from cells using RIPA lysis buffer (Thermo Fisher Scientific) and Halt protease inhibitor cocktail (Thermo Fisher Scientific). Protein concentration was measured using the Pierce BCA protein assay kit (Thermo Fisher Scientific) and cell lysates containing 20 µg of total protein were loaded and separated in 4-20% TGX precast SDS-PAGE gels (Bio-Rad, Hercules, CA) followed by transfer onto PVDF membranes (Bio-Rad). Immunoblot analyses were carried out as previously described (19). Mouse monoclonal antibody against GFP (1:1000, Santa Cruz, Dallas, Texas) and HRP-conjugated mouse monoclonal antibody against β-actin (1:50,000, Sigma-Aldrich, St. Louis, MO) were used for the assay.

### Live Imaging and Immunofluorescent Staining

Cells were seeded onto a Lab-Tek chambered coverslip (Sigma-Aldrich) and transfected with empty vector pEGFP-N1 or pEGFP-N1-ORF8 plasmids. GFP-live fluorescent images were captured and merged with differential interference contrast (DIC) bright-field images using a Leica Thunder microscope system (Wetzlar, Germany). For immunofluorescent staining, cells seeded onto Lab-Tek removable chamber slides were transfected with plasmids and Poly(I:C) as described above, then fixed using 4% paraformaldehyde (Thermo Fisher Scientific), permeabilized with 0.1% Triton X-100, and blocked with PBS buffer containing 2% normal goat serum (Vector Labs, Burlingame, CA) and 1% bovine serum albumin (Sigma-Aldrich). The slides were incubated at 4°C overnight with chicken anti-GFP primary antibody (1:500; Aves Lab, Tigard, OR). After incubation, slides were washed with PBS and incubated for 1 hour at room temperature in the dark with goat anti-chicken IgY (H+L) antibody labeled with Alexa Fluor 488 (1:250; Thermo Fisher Scientific). Finally, slides were washed with PBS and mounted with 4’,6-diamidino-2-phenylindole (DAPI)-contained mounting solution (Vector Labs). Slides were reviewed under a Leica Thunder microscope system and further processed by Adobe Photoshop software v21 (Adobe Systems Inc., San Jose, CA). For image quantification, 8-14 random images were acquired, and approximately 100-200 GFP-positive cells were counted from each group. To further confirm the subcellular distribution of ORF8^SARS-CoV-2^, cells were transfected with pcDNA3.1-ORF8-Flag construct and the transfected cells were processed for immunofluorescent staining with mouse anti-Flag M2 antibody (1:500; Sigma).

### Cell Proliferation Assay

Cells were plated in 24-well plates and co-transfected with plasmids with or without Poly(I:C) as described above. Cell proliferation was assessed 24 hours after transfection. In brief, cells were trypsinized and mixed with an equal volume of 0.4% trypan blue stain, then subjected to cell counting using a Countess 3 automated cell counter (Thermo Fisher Scientific). All experiments were conducted in triplicate.

### Flow Cytometry and Cell Cycle Analysis

To assess transfection efficiency, cells were plated in 6-well plates and transfected with plasmids. Cells were collected 24 hours post-transfection and stained with cell viability dye FVD-eFluor-506 (Thermo Fisher Scientific) at 4°C for 30 min, then subjected to flow cytometry analysis using a BD FACSymphony A5 Cell Analyzer (Indianapolis, IN). The GFP-positive live cell population was calculated by FlowJo (FlowJo LLC, Ashland, OR). For cell cycle analysis, cells were collected and washed twice with PBS, then resuspended in 1 mL PBS and fixed by adding 4 mL ice-cold absolute ethanol at -20°C overnight. After fixation, cells were washed and counter-stained with a PBS-buffered solution containing 0.1% Triton X-100, 3 µM propidium iodide (PI, Thermo Fisher Scientific), and 0.5 mg/mL RNase A (Sigma Aldrich) at 4°C for 30 min. Cell cycle distribution was examined using a BD FACSymphony A5 Cell Analyzer, and the proportion of cells in the G0−G1, S, and G2−M phases was determined using FlowJo software. All experiments were performed in triplicate.

### RNA Extraction and Quantitative Real-Time PCR (RT-qPCR) Analysis

Total RNA was extracted from cells using Trizol (Thermo Fisher Scientific) and purified by Zymo RNA Clean & Concentrator kit (Zymo, Irvine, CA). RNA was quantified with the Nanodrop (Agilent Technologies, Santa Clara, CA) and single-strand cDNA was generated using the iScript cDNA synthesis kit (Bio-Rad). RT-qPCR was performed using SYBR Green PowerUp PCR Universal Mastermix (Thermo Fisher Scientific) and the QuantStudio™ 6 real-time PCR system (Thermo Fisher Scientific), all according to the manufacturer’ manuals. The fold change in expression levels of target genes was calculated using the 2^−ΔΔCT^ method, with *GAPDH* as the endogenous reference. PCR reactions were run in duplicate for each sample. Primers used for RT-qPCR were synthesized by Integrated DNA Technologies and are listed in **Supplementary Table 1**. All experiments were repeated at least three times.

### *In Silico* Analysis

The ORF8^SARS-CoV-2^ sequence was retrieved from NCBI RefSeq MN908947 and submitted to online bioinformatics prediction tool PASTA2 (http://protein.bio.unipd.it/pasta2/). The propensity of ORF8 to form aggregates was assessed based on parallel aggregation probability and free energy.

### Statistical Analysis

All experiments were performed at least twice with triplicate samples. Statistical analysis was performed with GraphPad Prism 8 (GraphPad Software, San Diego, CA). Data are presented as mean ± s.d. and the statistical significance was assessed with either student’s *t*-test or one-way analysis of variance (ANOVA) followed by Fisher’s least significant difference post-hoc test. *p*<0.05 was considered statistically significant.

## Results

### Establishment of a Plasmid Construct to Study the Function of ORF8^SARS-CoV-2^ in Human Lung Epithelial Cells *In Vitro*

**Figure 1:**
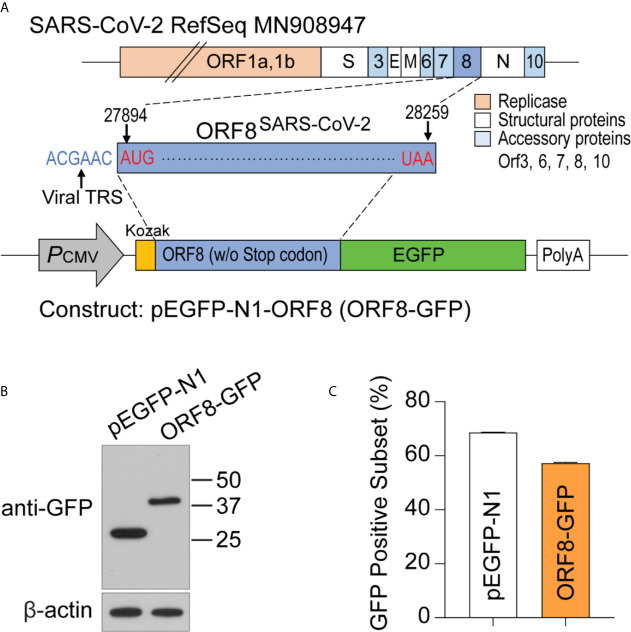
Generating a plasmid construct to study the function of ORF8^SARS-CoV-2^ in human A549 lung epithelial cells. **(A)** Cloning strategy to generate the pEGFP-ORF8^SARS-CoV-2^ (ORF8-GFP) construct. **(B)** Verification of ORF8-GFP expression in A549 lung epithelial cells transfected with pEGFP-ORF8^SARS-CoV-2^ by immunoblot analysis using anti-GFP antibody. Whole cell lysates were collected from cells transfected with empty vector (pEGFP-N1; EV) or ORF8-GFP at 24 hours post-transfection. **(C)** Characterization of the transfection efficiency of ORF8-GFP in A549 lung epithelial cells using FACS analysis. Cells were subjected to flow cytometry 24 hours after transfection with pEGFP-N1 (EV) or ORF8-GFP. The experiments were repeated twice with triplicate samples.

ORF8^SARS-CoV-2^ is an accessory protein encoded by the SARS-CoV-2 genome. This protein has been detected in sera of COVID-19 patients (16, 17), suggesting that ORF8^SARS-CoV-2^ is expressed in SARS-CoV-2 virus-infected cells. However, the function and pathobiological features of ORF8^SARS-CoV-2^ are largely unknown. To study ORF8^SARS-CoV-2^ biology, we prepared a plasmid, pEGFP-ORF8^SARS-CoV-2^, by inserting the full-length sequence of ORF8^SARS-CoV-2^ at the N-terminus of the enhanced green fluorescent protein (eGFP) of the pEGFP-N1 mammalian expression vector **(** **Figure 1A** **)**. After transfecting the pEGFP-ORF8^SARS-CoV-2^ plasmid into A549 cells, expression of eGFP-tagged ORF8^SARS-CoV-2^ protein (42 kDa) was confirmed by immunoblot with anti-GFP. eGFP (27 kDa) was expressed in the A549 cells transfected with pEGFP-N1 empty vector (EV) **(** **Figure 1B** **)**. When examined by flow cytometry analysis, approximately 60% of A549 cells were GFP-positive 24 h after transfection with either pEGFP-N1 or pEGFP-ORF8^SARS-CoV-2^ constructs **(** **Figure 1C** **)**, suggesting significant uptake of plasmids by A549 cells. This *in vitro* system was used to study the function of ORF8^SARS-CoV-2^ in human lung epithelial cells.

### ORF8^SARS-CoV-2^ Forms Intracellular Aggregates in Human Lung Epithelial Cells

**Figure 2:**
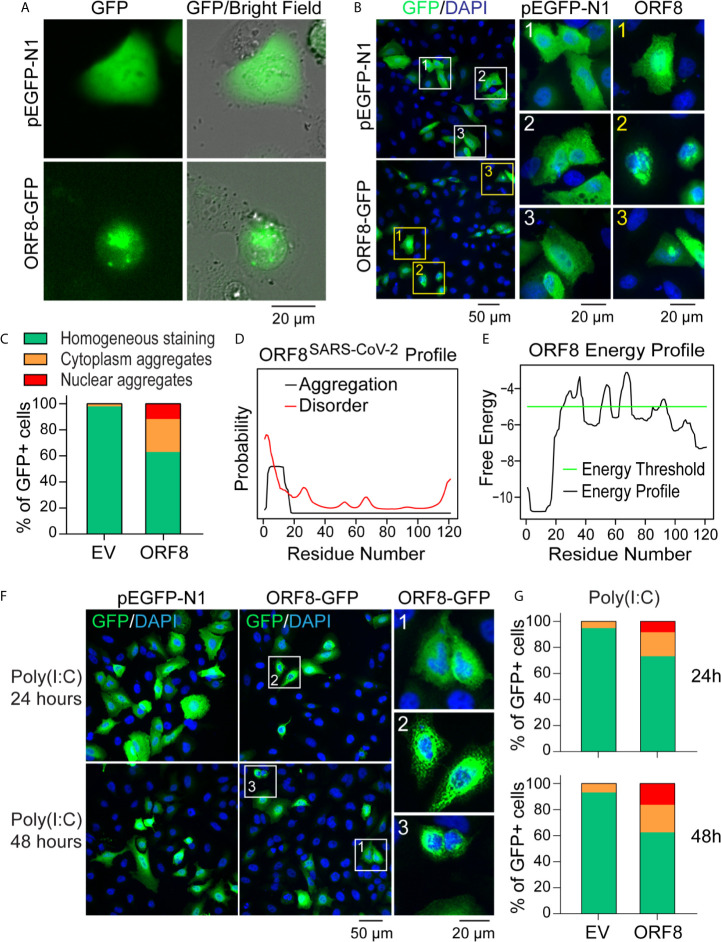
ORF8^SARS-CoV-2^ forms intracellular aggregates in human lung epithelial cells. **(A-C)** Aggregation of ORF8^SARS-CoV-2^ protein in A549 cells viewed by fluorescent microscopy. Human lung epithelial A549 cells were transfected with pEGFP-N1 (empty vector; EV) or pEGFP-ORF8^SARS-CoV-2^. After 24 h, fluorescent microscopy was performed on either live cells **(A)** or paraformaldehyde-fixed cells that were stained with anti-GFP antibody **(B)**. In the anti-GFP antibody-stained cells, quantitative analysis of nuclear-cytoplasmic distribution pattern of aggregated ORF8^SARS-CoV-2^ was performed **(C)**. **(D, E)** Prediction of aggregation based on the ORF8^SARS-CoV-2^ protein primary structure using the PASTA 2.0 algorithm (http://protein.bio.unipd.it/pasta2/). Aggregation disorder profile **(D)** and aggregation-free energy profile **(E)** showing that N-terminal residues 1-18 have the lowest aggregation free energy and thus is the most aggregation-stabilizing region underlying the propensity for ORF8^SARS-CoV-2^ aggregates formation. **(F, G)** The intracellular distribution pattern of ORF8^SARS-CoV-2^ aggregates is not affected by a poly (I:C)-induced inflammatory response. A549 cells were treated with poly (I:C) during transfection with pEGFP-N1 (EV) or pEGFP-ORF8^SARS-CoV-2^. After the indicated times, cells were fixed with paraformaldehyde followed by immunofluorescent staining with anti-GFP antibody. The stained cells were viewed by fluorescent microscopy **(F)** and processed for analysis of nuclear-cytoplasmic distribution pattern of ORF8^SARS-CoV-2^ aggregates **(G)**. 1 indicates homogenous distribution; 2, cytoplasmic aggregates; and 3, nuclear aggregates.

Because SARS-CoV-2 targets human lung epithelial cells (20, 21), we first visualized the distribution of the SARS-CoV-2 ORF8 accessory protein in A549 cells using the pEGFP-ORF8^SARS-CoV-2^ plasmid construct. Using live fluorescent imaging, we found that pEGFP-N1-transfected A549 cells exhibited homogeneous GFP fluorescence **(** **Figure 2A** **)**. In contrast, distinctive fluorescent particles were seen in A549 cells transfected with the pEGFP-ORF8^SARS-CoV-2^ construct **(** **Figure 2A** **)**, suggesting that ORF8^SARS-CoV-2^ forms aggregates in lung epithelial cells. In addition, immunofluorescence staining revealed that ectopically expressed ORF8^SARS-CoV-2^-GFP protein was either homogenously distributed in A549 cells or formed aggregates in cytoplasmic and nuclear compartments of the cells **(** **Figure 2B** **)**. Image quantification revealed a higher ratio of intracellular aggregates in pEGFP-ORF8^SARS-CoV-2^-transfected cells compared to cells transfected with pEGFP-N1 empty vector **(** **Figure 2C** **)**. To exclude the possibility that eGFP fusion to C-terminal of ORF8^SARS-CoV-2^ could affect the subcellular distribution of ORF8^SARS-CoV-2^, cells were transfected with pcDNA3.1-ORF8-Flag construct followed by immunofluorescent staining with anti-Flag antibody. Using this approach, we confirmed a similar subcellular expression pattern of ORF8^SARS-CoV-2^ **(** **Supplementary Figure 1** **)**, suggesting that ectopic expression of ORF8^SARS-CoV-2^ forms cytoplasmic and nuclear aggregates in the lung epithelial cells without contribution of Tag proteins. Using *in silico* prediction (http://protein.bio.unipd.it/pasta2/) (22), we found that ORF8^SARS-CoV-2^ possesses an intrinsic aggregation characteristic at its N-terminal residues 1-18 **(** **Figures 2D, E** **)**, with a total of 20 potential aggregations predicted with the best energy -10.07 **(** **Supplementary Table 2** **)**.

We next examined whether activation of antiviral immune response affects the formation of ORF8^SARS-CoV-2^ aggregates in lung epithelial cells. A549 cells were stimulated with Poly(I:C) to mimic the activation of cells by viral infection. As expected, treatment with Poly(I:C) profoundly induced the expression of various antiviral genes in A549 cells **(** **Supplementary Figure 2** **)**, indicating activation of the antiviral immune response. Next, cells were co-transfected with a mixture of plasmids and Poly(I:C) to examine the formation of ORF8^SARS-CoV-2^ aggregates in lung epithelial cells under a Poly(I:C)-mimicked antiviral microenvironment. Immunostaining analysis did not reveal any change in the distribution pattern of ORF8^SARS-CoV-2^ aggregates in poly(I:C)-treated cells **(** **Figures 2F, G** **)**. These data suggest that ORF8^SARS-CoV-2^ forms aggregates in both the cytosol and nucleus of lung epithelial cells, independent from inflammatory events activated by viral infection.

### Expression of ORF8^SARS-CoV-2^ Does Not Affect Lung Epithelial Cell Proliferation and Cell Cycle Activity

**Figure 3:**
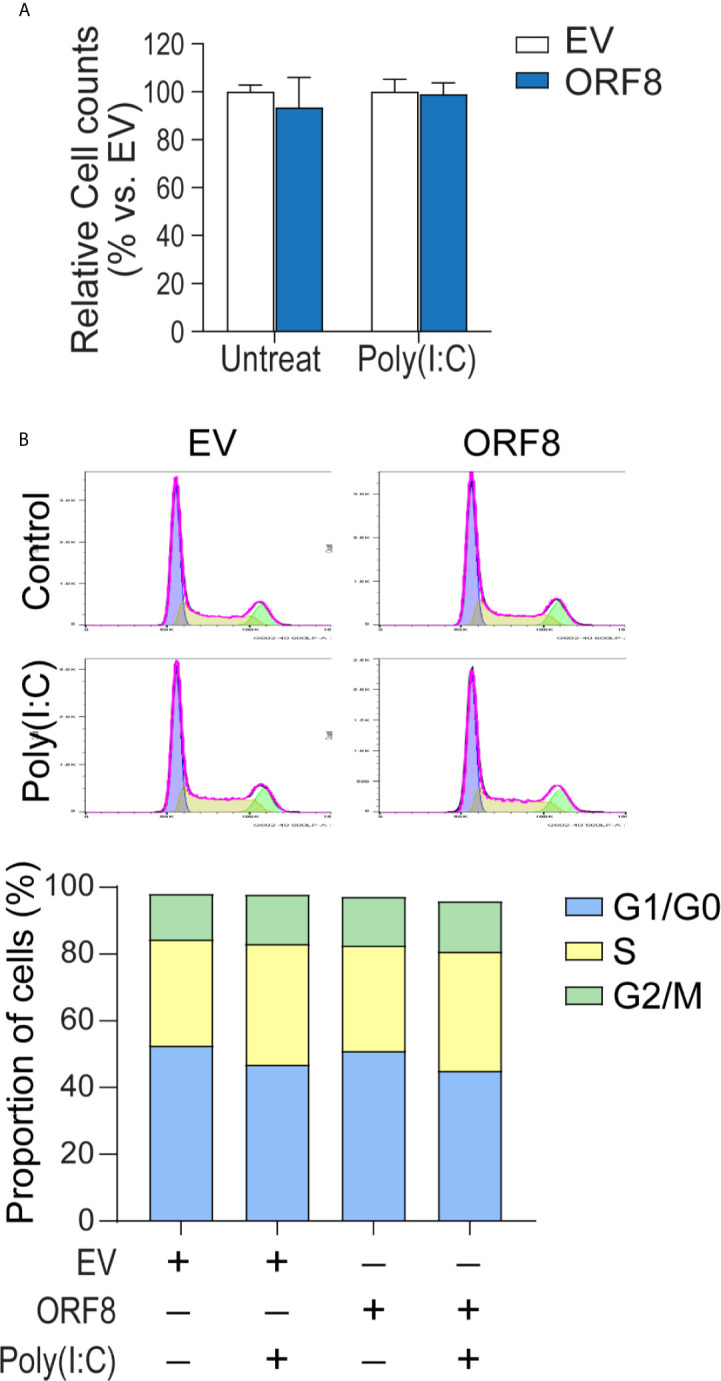
Expression of ORF8^SARS-CoV-2^ does not affect lung epithelial cell proliferation or cell cycle progression. Human A549 lung epithelial cells were transfected with pEGFP-N1 (empty vector, EV) or pEGFP-ORF8^SARS-CoV-2^ and treated with or without poly (I:C). At 24 hours post-transfection, cells were processed for *in vitro* proliferation assay **(A)** and flow cytometry-based cell cycle analysis **(B)**. n = 3 for each group.

Intracellular protein aggregates can be toxic, causing injury or death to cells (23, 24). Thus, we next examined whether lung epithelial cell growth is affected by ectopic ORF8^SARS-CoV-2^ expression. Cell numbers of pEGFP-N1-transfected and pEGFP-ORF8^SARS-CoV-2^-transfected A549 cells were compared in the presence or absence of poly(I:C). Cells transfected with pEGFP-ORF8^SARS-CoV-2^ exhibited a similar growth rate to empty vector-transfected cells when cultured with or without poly(I:C) **(** **Figure 3A** **)**.

It has been shown that the interference with cell cycle progression is a common host response upon viral infection (25, 26). Thus, we investigated the influence of ORF8^SARS-CoV-2^ expression on cell cycle progression in A549 cells using flow cytometry analysis. We noted that cell cycle phases (G1/G0, S, G2/M) in pEGFP-ORF8^SARS-CoV-2^-transfected cells were similar to those in pEGFP-N1-transfected cells, regardless of poly(I:C) stimulation **(** **Figure 3B**), suggesting that expression of ORF8^SARS-CoV-2^ does not change cell cycle status. Therefore, neither expression of ORF8^SARS-CoV-2^ nor formation of ORF8^SARS-CoV-2^ protein aggregates influenced lung epithelial cell proliferation and cell cycle progression.

### ORF8^SARS-CoV-2^ Inhibits Baseline Expression of Several Antiviral Immunity-Associated Molecules in the Lung Epithelial Cells

**Figure 4:**
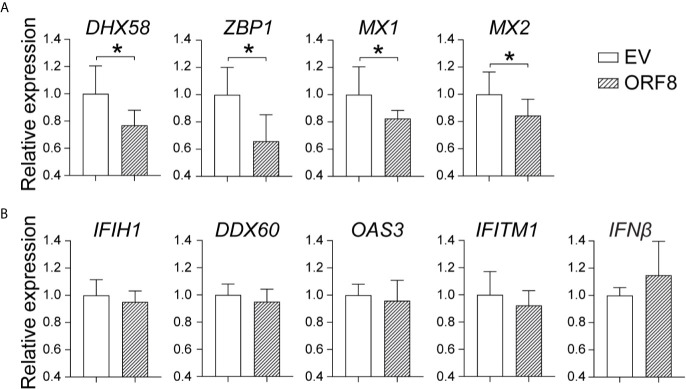
Characterization of the role of ORF8^SARS-CoV-2^ on expression of antiviral immunity-associated genes in lung epithelial cells. A549 cells were transfected with pEGFP-N1 (empty vector, EV) or pEGFP-ORF8^SARS-CoV-2^. At 24 hours post-transfection, cells were processed for RNA extraction and RT-qPCR to determine the expression levels of indicated immune response genes. **(A)** ORF8-inhibited genes. **(B)** Non-responsive genes. n = 3-5, *p < 0.05 was considered as statistically significant.

To further determine the effect of ORF8^SARS-CoV-2^ on the antiviral immune response, we examined whether ORF8^SARS-CoV-2^ affects expression of viral infection-associated innate immune molecules (**Table 1**) in A549 cells. Using RT-qPCR, we found that ectopic expression of ORF8^SARS-CoV-2^ significantly inhibited expression of some members of the IFN-stimulated gene (ISG) family including *ZBP1* (an innate sensor of viral infection), *MX1* and *MX2* (host restriction factors of viral replication), and *DHX58* (a critical molecule in the RIG-I cytosolic pattern recognition receptor pathway) in A549 cells **(** **Figure 4A**). However, expression of ORF8^SARS-CoV-2^ did not influence the expression of *IFNβ* and several other IFN signature genes including *IFIH1*, *DDX60*, *OAS3*, and *IFITM1* **(** **Figure 4B** **)**. To determine whether ORF8^SARS-CoV-2^ could affect the basal-level expression of these ISG-associated genes in other type of cells, we further transfected HEK293 embryonic kidney epithelial cells with pEGFP-ORF8^SARS-CoV-2^. We revealed significant decrease in expression of *OAS3* and *IFITM1* in ORF8^SARS-CoV-2^-transfected HEK293 cells comparing to the control cells **(** **Supplementary Figure 3** **)**. Interestingly, the expression of these two genes in A549 cells was not affected by ectopic ORF8^SARS-CoV-2^ **(** **Figure 4B** **)**. These data suggest that ORF8^SARS-CoV-2^ selectively disrupts expression of several innate immunity-associated molecules without affecting IFN signaling in a cell type specific manner.

**Table 1:** Viral infection-associated innate immune molecules.

Gene Symbol	Full Name	Function
IFNβ	interferon beta	IFNβ is type I class of interferon, an important cytokine for defense against viral infections (27–29)
IFIH1	interferon induced with helicase C domain 1	IFIH1 encodes MDA5, an intracellular sensor of viral RNA, thus triggering the host cell innate immune response (30, 31)
DHX58	DExH-box helicase 58	DHX58 is a critical molecule in the RIG-I cytosolic pattern recognition receptor pathway (32–34)
DDX60	DExD/H-box helicase 60	DDX60 encodes a DEXD/H box RNA helicase that functions as an antiviral factor and promotes RIG-I-like receptor-mediated signaling (35, 36)
ZBP1	Z-DNA binding protein 1	ZBP1 is an innate sensor of viral infection (37)
OAS3	2’-5’-oligoadenylate synthetase 3	OAS3 is an interferon-stimulated gene and activates RNase L, which is involved in the inhibition of cellular protein synthesis and viral infection resistance (38, 39)
MX1	MX dynamin like GTPase 1	MX1 is an interferon-stimulated gene that participates in the cellular antiviral response by antagonizing the replication process of several different RNA and DNA viruses (40)
MX2	MX dynamin like GTPase 2	MX2 is an interferon-induced post-entry inhibitor of viral infection that acts by targeting the viral capsid to affect the nuclear uptake and/or stability of virus replication complex (41, 42)
IFITM1	Interferon-induced transmembrane protein 1	IFITM1 is an interferon-stimulated gene and functions through preventing infection before a virus can traverse the lipid bilayer of the cell (43, 44)

### ORF8^SARS-CoV-2^ Attenuates A549 Cell Production of Antiviral Molecules Induced by IFNγ But Not by IFNβ

**Figure 5:**
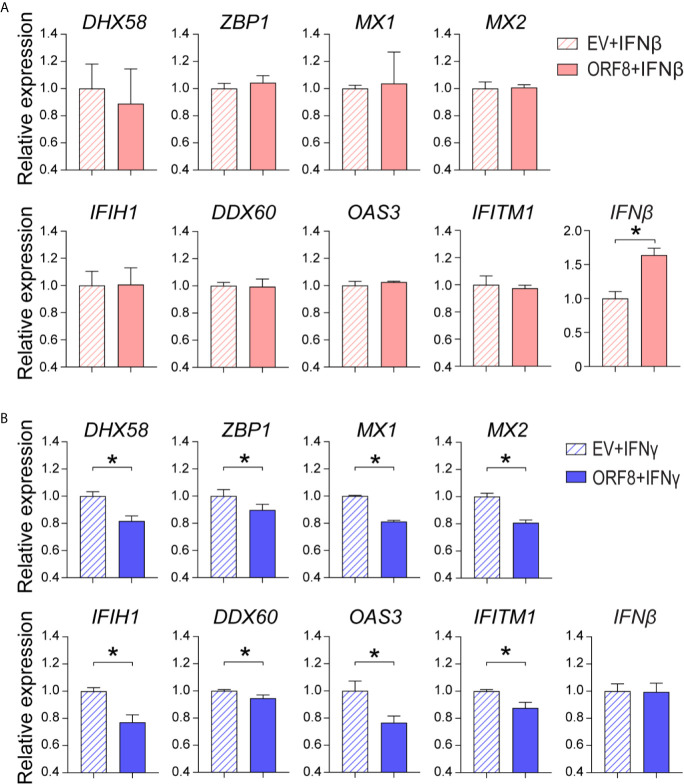
Characterization of the role of ORF8^SARS-CoV-2^ on regulation of type I and II interferon (IFN)-induced expression of antiviral molecules in human lung epithelial cells. Human A549 lung epithelial cells were transfected with pEGFP-N1 (empty vector, EV) or pEGFP-ORF8^SARS-CoV-2^. The transfected cells were treated with medium containing IFNβ (100 ng/mL) **(A)** or IFNγ (100 ng/mL) **(B)** for 24 h followed by RNA extraction and RT-qPCR analysis of expression of the indicated genes. n = 3-5. *p < 0.05 was considered as statistically significant.

IFNγ and IFNβ play critical roles in the regulation of the antiviral immune response (27, 28). Thus, we examined whether ORF8^SARS-CoV-2^ affects IFNγ and/or IFNβ-induced ISG expression in A549 cells. First, we found that both IFNγ and IFNβ markedly induced expression of several antiviral molecules **(** **Supplementary Figures 4A, B** **)**. Expression of ORF8 did not change IFNβ-induced expression of most antiviral molecules assessed, but did increase IFNβ-induced endogenous *IFNβ* expression in lung epithelial cells **(** **Figure 5A**). In contrast, expression of ORF8 in lung epithelial cells significantly decreased IFNγ-induced antiviral molecules **(** **Figure 5B**), suggesting that expression of ORF8^SARS-CoV-2^ inhibits IFNγ-mediated antiviral immunity. Together, it appears that ORF8^SARS-CoV-2^ affects the IFNγ- but not IFNβ-induced host antiviral response in lung epithelial cells.

## Discussion

In this study, we characterized the biological and immunobiological effects of the SARS-CoV-2 ORF8 accessory protein in lung epithelial cells. Specifically, we found that the N-terminal sequence of ORF8^SARS-CoV-2^ is composed of features that favor the formation of protein aggregates. We showed that ectopic expression of ORF8^SARS-CoV-2^ results in formation of cytoplasmic and nuclear ORF8 protein aggregates in lung epithelial cells, forming structures that can profoundly influence cell processes (12, 23). Interestingly, expression of ORF8^SARS-CoV-2^ did not affect lung epithelial cell proliferation and cell cycle progression regardless the activation of antiviral responses in the cells. Rather, ORF8^SARS-CoV-2^ expression was found to attenuate the IFNγ- but not IFNβ-mediated antiviral gene expression in lung epithelial cells. Together, our study suggests that ORF8^SARS-CoV-2^ forms aggregates in lung cells that then impair the molecular machinery of the antiviral immune response.

Evidence suggests that host cells form insoluble aggregates/inclusions during viral infections (23), which then have various functions in cells. For example, viral infection-associated protein aggregates can form protective structures that facilitate viral escape from cellular degradation machinery (23, 45). Viruses can also utilize aggregates to promote viral replication, gene translation, and intra- and intercellular transportation (23, 45). In addition, protein aggregates and misfolded proteins themselves can cause host cell death by inducing ER stress and the unfolded protein response (UPR) (12). Previously, SARS-CoV ORF8b was found to form insoluble intracellular aggregates through its C-terminus; aggregated ORF8b caused the death of epithelial cells through induction of ER stress and subsequent activation of the autophagy-lysosome pathway (12). In the present study, we detected the intracellular aggregation of ORF8^SARS-CoV-2^ in lung epithelial cells using a nonvirus-infection approach, but we did not observe cytotoxic effects of ORF8^SARS-CoV-2^ aggregates in the cells. Thus, we speculate that ORF8^SARS-CoV-2^ aggregates might not overwhelm cellular homeostatic mechanisms in our experimental settings *in vitro*.

The mechanism underlying formation of ORF8^SARS-CoV-2^ aggregates is not clear. Evidence shows that typical residues and regions in ORF proteins of coronavirus play an important role in formation of protein aggregates. For instance, Ng and Liu previously revealed that coronavirus IBV ORF 1a forms aggregates, which seems to be mediated the presence of a hydrophobic domain downstream of the 3C-like proteinase-encoding region in this ORF protein (46). Shi et al.** recently reported that ORF8b of SARS-CoV forms intracellular aggregates dependent on a valine at residue 77 of this ORF protein (12). Using a bioinformatic approach, we noted that N-terminal residues 1-18 of ORF8^SARS-CoV-2^ may affect the aggregation propensity of this protein. Further experimental analysis is required to clarify exact roles of these residues in formation of intracellular aggregates in ORF8^SARS-CoV-2^ expressing cells.

Our findings are consistent with a growing body of evidence showing that accessory proteins of pathogenic CoVs play a role in disrupting antiviral immune responses (6, 7, 47). For instance, SARS-CoV ORF3a was found to promote ubiquitination and lysosomal pathway-dependent degradation of IFNAR1, which results in attenuation of the type I IFN response (48). SARS-CoV ORF8b and 8ab were reported to act as IFN antagonists through mediating ubiquitin-dependent rapid degradation of IRF3 (49). Recently, two independent studies demonstrated that SARS-CoV-2 ORF6 is a potent IFN antagonist that inhibits the IFNβ promoter, ISRE, and NF-κB element in IFN-stimulated genes (ISGs) (47, 50). Furthermore, Miorin et al. (51) reported that SARS-CoV-2 ORF6 inhibits IFN signaling through blocking STAT nuclear import. Collectively, it appears that accessory proteins encoded by pathogenic CoV genomes influence immunological activities in the host cells.

SARS-CoV-2 infects human lung epithelial cells which are increasingly recognized as an important immunological barrier. In the present study, we found that ORF8^SARS-CoV-2^ selectively attenuates the basal expression of several ISGs including *ZBP1*, *MX1* and *MX2*, and *DHX58* in the lung epithelial cells. Among these genes, ZBP1 and DHX58 are reported to play an important role in sensing viral pathogens (32–34, 37, 52), whereas MX1 and MX2 are critical antiviral gatekeepers in cells (40, 53). Interestingly, IFN production is impaired in COVID-19 patients (54). Thus, our findings suggest that ORF8^SARS-CoV-2^ attenuates basal-level expression of diverse viral-sensing molecules; the lack of IFN signaling subsequently facilitates replication of SARS-CoV-2 in the host cells.

Evidence shows that signaling cascades activated by IFNα/β are of particular importance in the host response to viral pathogens (27–29). Recently, several investigators studied the role of ORF8^SARS-CoV-2^ in the regulation of the type I IFN-associated antiviral immune responses, though the conclusions drawn from these studies remain controversial. For example, Li et al. (47) reported that ORF8^SARS-CoV-2^ is a potent IFN antagonist that delays the release of IFNs through inhibition of IFNβ promoter activity in HEK 293T cells. Yuen et al. (50) found that ORF8^SARS-CoV-2^ does not suppress primary IFN production and signaling in 293FT cells. Here, we showed that expression of ORF8^SARS-CoV-2^ promotes the autocrine effect of IFNβ on its own gene expression but does affect IFNβ-mediated induction of antiviral ISGs family genes in lung epithelial cells. Taken together, our data, combined with findings from other investigators, suggest that ORF8^SARS-CoV-2^ affects type I IFN signaling in a cell-type dependent manner *via* a complex mechanism.

In addition to IFNα/β, emerging evidence shows that IFNγ carries out unique functions in antiviral immunity (55). For example, NK cells or innate lymphoid type I cells produce IFNγ to modulate the function of surrounding innate immune cells, including macrophages and dendritic cells, which subsequently regulate the antiviral immune response (27, 55). In the present study, we showed that IFNγ profoundly induces the expression of various antiviral molecules in lung epithelial cells, suggesting that IFNγ enhances the innate immune function in the lung epithelial barrier. Furthermore, we found for the first time that expression of ORF8^SARS-CoV-2^ significantly inhibits IFNγ-mediated upregulation of antiviral gene expression, suggesting that ORF8^SARS-CoV-2^ hinders IFNγ-regulated antiviral immunity. The mechanism underlying the blockage of IFNγ activity by ORF8^SARS-CoV-2^ needs to be studied in the future.

Upon virus infection, the host cells initiate and launch innate antiviral immune responses. In present study, we found ORF8^SARS-CoV-2^ attenuates basal-level expression of diverse viral-sensing molecules and suppresses IFNγ-regulated antiviral immunity in cultured A549 human lung epithelial cells. However, it remains unclear whether ORF8^SARS-CoV-2^ hinders host cells antiviral immunity in SARS-CoV-2 infected cells. To firmly elucidate the role of ORF8^SARS-CoV-2^ in coronavirus pathogenesis by targeting ISGs of lung epithelial cells, it would be of interest to further study cell biological and immunological functions of ORF8^SARS-CoV-2^ in SARS-CoV-2 infected cells using mutagenesis and RNAseq approaches in the future. In addition, our data suggest that formation of ORF8^SARS-CoV-2^ aggregates in cells takes place without active SARS-CoV-2 infection, which may affect cell functions. Thus, future studies are needed to determine whether persistent ORF8^SARS-CoV-2^ expression occurs in COVID-19 patients after recovering from SARS-CoV-2 infection. If so, it is possible that prolonged ORF8^SARS-CoV-2^ expression may play a pathogenic role in long-haul COVID-19 symptoms.

Collectively, our study suggests that ORF8^SARS-CoV-2^ displays several potent pathogenic activities in human lung epithelial cells including formation of intracellular aggregates, disruption of basal expression of multiple types of viral sensing molecules, and inhibition of IFNγ-induced antiviral gene expression. However, the link among these ORF8^SARS-CoV-2^-associated cell biological and immunological events is not clear. Furthermore, molecular mechanisms underlying these processes are of interest to study in the future, which will advance our knowledge in regarding the role of ORF8^SARS-CoV-2^ in pathogenesis of COVID-19 and development of novel therapeutic approaches for maintaining homeostasis of antiviral immunity in patients with COVID-19.

## Data Availability Statement

The original contributions presented in the study are included in the article/**Supplementary Material**. Further inquiries can be directed to the corresponding author.

## Author Contributions

HG and X-DT were involved in the overall design of experiments and interpretation of results. HG, SS, LW, and H-FB performed all experiments. HG and LW prepared figures. HG, SS, and X-DT wrote manuscript with input from all authors. XW, CD, and IP critically reviewed and commented on the work. HG and X-DT conceived and orchestrated the project. All authors contributed to the article and approved the submitted version.

## Funding

HG is a recipient of the COVID-19 Exploratory Springboard Award from the Stanley Manne Children’s Research Institute, the Ann & Robert H. Lurie Children’s Hospital of Chicago. X-DT is funded by US National Institutes of Health (NIH) grants (R01GM117628, R01GM122406, and R01DK123826) and US Department of Veterans Affairs Merit Review Award (I01BX001690). IP is funded by NIH grant R01DK116568. The funders had no role in study design, data collection and analysis, interpretation of data, decision to publish, or preparation of the manuscript.

## Conflict of Interest

The authors declare that the research was conducted in the absence of any commercial or financial relationships that could be construed as a potential conflict of interest.

## Abbreviations

SARS-CoV-2, severe acute respiratory syndrome coronavirus 2; COVID19, coronavirus disease 2019; ORF8, open reading frame 8; ISG, interferon-stimulated gene; IFNβ, interferon beta; IFNγ, interferon gamma; IFIH1, interferon induced with helicase C domain 1; DHX58, DExH-box helicase 58; DDX60, DExD/H-box helicase 60; ZBP1, Z-DNA binding protein 1; OAS3, 2’-5’-oligoadenylate synthetase 3; MX1, MX dynamin like GTPase 1; MX2, MX dynamin like GTPase 2; IFITM1, interferon-induced transmembrane protein 1.
