# Supplementary material for: SARS-CoV-2 ORF8 Forms Intracellular Aggregates and Inhibits IFNγ-Induced Antiviral Gene Expression in Human Lung Epithelial Cells
Source: Front Immunol. 2021 Jun 9;12:679482. doi: 10.3389/fimmu.2021.679482 (PMC8221109; doi:10.3389/fimmu.2021.679482)
Supplement: Supplementary file 1 [file DataSheet_1.pdf]

Supplementary Figures

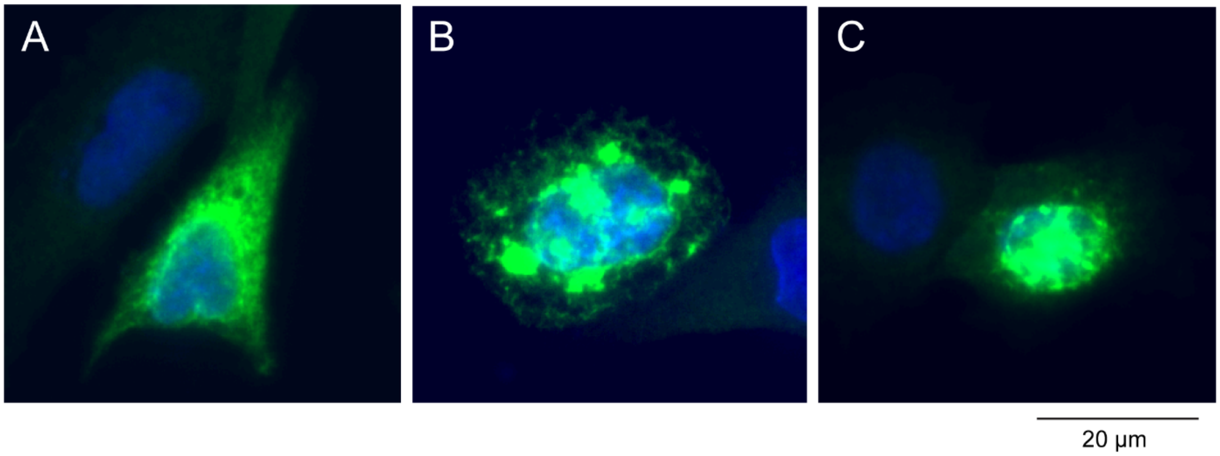

Supplementary Figure 1. ORF8$^{SARS\text{-}CoV\text{-}2}$ forms intracellular aggregates in human lung epithelial cells. A549 cells were transfected with pcDNA-ORF8-Flag construct. After 24 hours, cells were fixed with paraformaldehyde followed by immunofluorescent staining with anti-Flag M2 antibody. The stained cells were viewed by fluorescent microscopy. (A) Homogenous distribution; (B) cytoplasmic aggregates; and (C) nuclear aggregates.

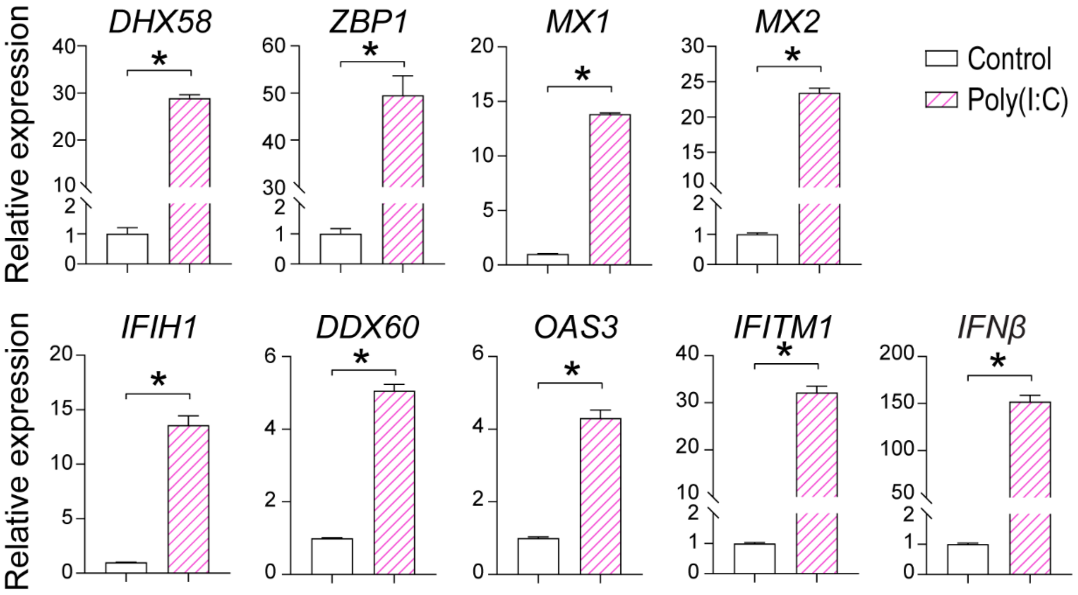

Supplementary Figure 2. Poly(I:C) profoundly induced expression of various antiviral genes in human lung epithelial cells. A549 cells seeded in 6-well plate were treated with Poly(I:C) (25 ng/well) for 24 h using a protocol described in the method section. At the end of treatment, cells were processed for RNA extraction and RT-qPCR analysis of expression of indicated genes. n=3-5, *p<0.05 was considered as statistically significant.

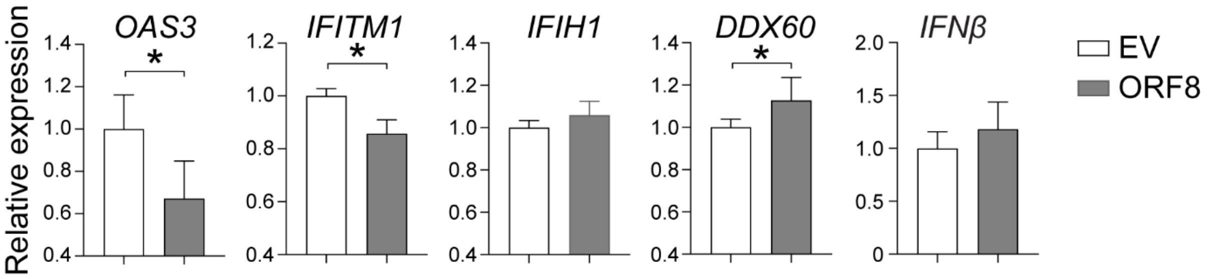

Supplementary Figure 3. Characterization of the effect of ORF8$^{SARS-CoV-2}$ on expression of antiviral immunity-associated genes in embryonic kidney epithelial cells. HEK293 cells were transfected with pEGFP-N1 (empty vector, EV) or pEGFP-ORF8$^{SARS-CoV-2}$. At 24 hours post-transfection, cells were processed for RNA extraction and RT-qPCR to measure the expression levels of indicated immune response genes. n=3-5, *p<0.05 was considered as statistically significant.

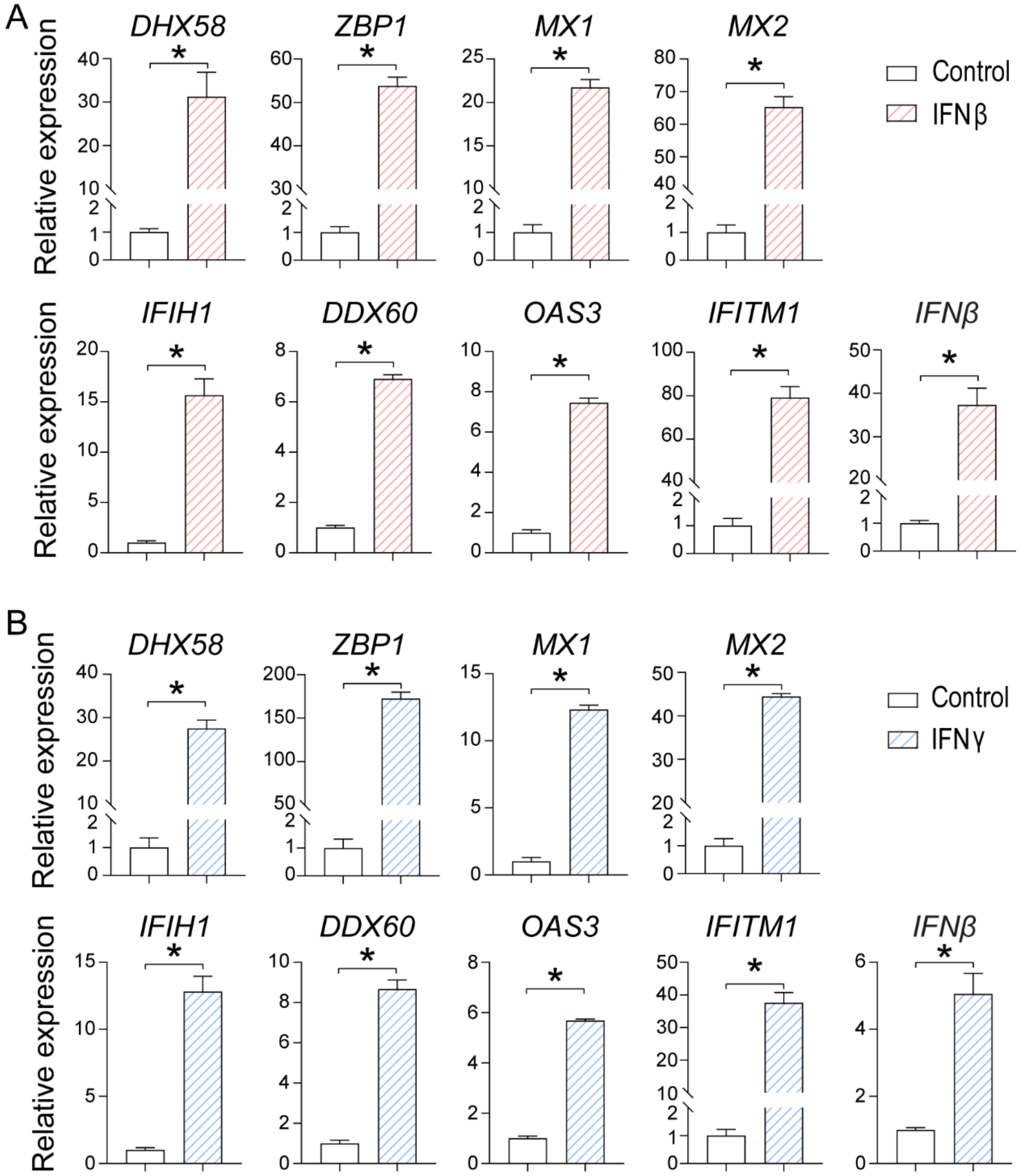

Supplementary Figure 4. Interferons robustly induced expression of antiviral genes in human lung epithelial cells. A549 cells were treated with **(A)** IFNβ (100 ng/mL) or **(B)** IFNγ (100 ng/mL) for 24 hours and processed for RNA extraction and RT-qPCR analysis of expression of indicated genes. n=3-5, *$p<0.05$ was considered as statistically significant.
