# Supplementary material for: SARS-CoV-2 ORF8 Forms Intracellular Aggregates and Inhibits IFNγ-Induced Antiviral Gene Expression in Human Lung Epithelial Cells
Source: Front Immunol. 2021 Jun 9;12:679482. doi: 10.3389/fimmu.2021.679482 (PMC8221109; doi:10.3389/fimmu.2021.679482)
Supplement: Supplementary file 2 [file DataSheet_2.pdf]

Supplementary Tables

Supplementary Table 1. Primer sequences used in this study

**Target Gene**	**Primer Name**	**Sequence (5'-3')**
GAPDH	GAPDH F	TGCACCACCAACTGCTTAGC
	GAPDH R	GGCATGGACTGTGGTCATGAG
IFIH1	IFIH1 F	GCTGAAGTAGGAGTCAAAGCCC
	IFIH1 R	CCACTGTGGTAGCGATAAGCAG
DHX58	DHX58 F	ATGACCACCTGGAGATGCCTGA
	DHX58 R	CATTGTAGCGCCTCAGGTGAAG
DDX60	DDX60 F	TTGTGCACTGGCATTCTCAT
	DDX60 R	TCAGCCTTGGTCTCGTGTGC
OAS3	OAS3 F	TCAGAAGCCCAGGCCTATCAT
	OAS3 R	CAACTTCACACAGCAGCCTTCA
ZBP1	ZBP1 F	GGGACACAGCAATGAGATGA
	ZBP1 R	TCCTGGACTGGGAATTCTTG
MX1	MX1 F	GGCTGTTTACCAGACTCCGACA
	MX1 R	CACAAAGCCTGGCAGCTCTCTA
MX2	MX2 F	ATGAATGTGGTGCGGAACCT
	MX2 R	AAGTCTTTCTGCCAGTCGGG
IFITM1	IFITM1 F	AGAAGATGCACAAGGAGGAACAT
	IFITM1 R	ATATGGTAGACTGTCACAGAGCCG
IFNβ	IFNB F	GTGGCAATTGAATGGGAGGC
	IFNB R	GTCTCATTCCAGCCAGTGCT
pcDNA3.1-ORF8-Flag construct	ORF8-Flag-F	TACCGAGCTCGGATCCGCCACCATGAAATTTCTTGTTTTCTTAGGAATCATC
	ORF8-Flag-R	GATATCTGCAGAATTCTTACTTGTCATCGTCGTCCTTGTAGTCGATGAAATCTAAAACAACACGAACGTC

Supplementary Table 2. List of predicted amyloidogenic peptides in ORF8$^{SARS\text{-}CoV\text{-}2}$ protein

Parallel Aggregates	Free Energy (Kcal/mol)	Pairing Segments (aa)
#1	-10.07	3-13
#2	-9.96	3-16
#3	-9.88	3-17
#4	-9.57	3-14
#5	-9.18	4-13
#6	-9.07	3-15
#7	-9.07	4-16
#8	-8.98	4-17
#9	-8.91	1-13
#10	-8.80	1-16
#11	-8.73	2-13
#12	-8.72	1-17
#13	-8.68	4-14
#14	-8.62	2-16
#15	-8.54	2-17
#16	-8.44	3-18
#17	-8.43	5-13
#18	-8.41	1-14
#19	-8.31	5-16
#20	-8.23	2-14

Identification of aggregation-prone fragments in ORF8$^{SARS\text{-}CoV\text{-}2}$ protein was done using the PASTA 2 prediction server (http://protein.bio.unipd.it/pasta2/). The list shows all aggregation prone peptides that possess a threshold of free energy < - 5, which are in ORF8$^{SARS\text{-}CoV\text{-}2}$ N-terminal.
